# Environmental factors influencing primary productivity of the forest-forming kelp *Laminaria hyperborea* in the northeast Atlantic

**DOI:** 10.1038/s41598-020-69238-x

**Published:** 2020-07-22

**Authors:** Dan A. Smale, Albert Pessarrodona, Nathan King, Michael T. Burrows, Anna Yunnie, Thomas Vance, Pippa Moore

**Affiliations:** 1 Marine Biological Association of the United Kingdom, The Laboratory, Citadel Hill, Plymouth, PL1 2PB UK; 2 UWA Oceans Institute and School of Biological Sciences, University of Western Australia, Crawley, WA 6009 Australia; 3 School of Ocean Sciences, Bangor University, Menai Bridge, Anglesey, LL59 5AB UK; 4 Scottish Association for Marine Science, Dunbeg, Oban, PA37 1QA Argyll UK; 5 PML Applications Ltd, Prospect Place, Plymouth, PL1 3DH UK; 6 Institute of Biological, Environmental and Rural Sciences, Aberystwyth University, Aberystwyth, SY23 3DA UK; 7 Centre for Marine Ecosystems Research, School of Natural Sciences, Edith Cowan University, Joondalup, WA 6027 Australia

**Keywords:** Biogeochemistry, Climate-change ecology, Ecosystem ecology, Population dynamics, Marine biology

## Abstract

Rates and drivers of primary productivity are well understood for many terrestrial ecosystems, but remain poorly resolved for many marine ecosystems, particularly those within in coastal benthic environments. We quantified net primary productivity (NPP) using two methods as well as carbon standing stock within kelp forests (*Laminaria hyperborea*) at multiple subtidal habitats in the United Kingdom (UK). Study sites spanned 9° in latitude and encompassed a gradient in average temperature of ~ 2.5 °C. In addition to temperature, we measured other factors (e.g. light intensity, water motion, nutrients, sea urchin density) that may influence productivity. Although estimates of NPP were highly variable between sites, ranging from 166 to 738 g C m^-2^ yr^-1^, our study-wide average of 340 g C m^-2^ yr^-1^ indicated that *L. hyperborea* forests are highly productive. We observed clear differences between NPP and carbon standing stock between our cold northernmost sites and our warm southernmost sites, with NPP and standing stock being around 1.5 and 2.5 times greater in the northern sites, respectively. Ocean temperature was identified as a likely driver of productivity, with reduced NPP and standing stock observed in warmer waters. Light availability was also strongly linked with carbon accumulation and storage, with increased light levels positively correlated with NPP and standing stock. Across its geographical range, total NPP from *L. hyperborea* is estimated at ~ 7.61 Tg C yr^-1^. This biomass production is likely to be important for local food webs, as a trophic subsidy to distant habitats and for inshore carbon cycling and (potentially) carbon sequestration. However, given the strong links with temperature, continued ocean warming in the northeast Atlantic may reduce primary productivity of this foundation species, as optimal temperatures for growth and performance are surpassed.

## Introduction

Primary productivity underpins most food webs and ecosystems on Earth, and as such, understanding rates, trends and drivers of primary production by autotrophs is a fundamental goal of ecology^1,2^. Net primary productivity (NPP; gross primary productivity minus energy required for respiration and maintenance) is as important in the marine realm as it is on land, yet current understanding of rates of NPP is comparatively poor for many marine ecosystems and habitats^3,4^. Even so, the most reliable available estimates suggest that ocean-based NPP is significant, accounting for ~ 50% of NPP on Earth^5^, with approximately 90% of that contribution attributed to open ocean phytoplankton, 10% attributed to coastal macrophytes (e.g. seagrass meadows, macroalgal beds) and 1% to microphytobenthos^3,6,7^. Despite making a modest contribution to total ocean-based NPP and covering just ~ 2% of the area of the global ocean, coastal macrophytes play a disproportionately important role in the oceanic carbon cycle as they fix and sequester ~ 50% of all carbon stored in marine sediments^4,7,8^.

Macroalgal habitats (e.g. kelp forests and seaweed beds) are the most extensive and productive coastal ecosystems at a global scale^4,9^. Carbon cycling here is characterised by rapid biomassturnover, which results in large amounts of carbon entering the marine environment as detritus^9,10^. The flow of organic matter from macroalgal habitats represents a significant trophic subsidy for bacteria, detritivores and filter feeders^11,12^, while some emerging evidence suggests that a significant proportion of the carbon they assimilate may be stored in long-term reservoirs and, as such, could be important for natural sequestration^9,13,14^. However, quantifying carbon stores and flows through macroalgal habitats remains challenging, as technologies commonly used to measure production on land or the open ocean (e.g. satellite monitoring) have limited utility in most submerged benthic environments. As such, estimates of macroalgal NPP obtained over large spatial scales and along environmental gradients are needed to elucidate the role of macroalgal habitats in the oceanic carbon cycle and natural carbon sequestration^8,9^.

The large kelp *Laminaria hyperborea* functions as the dominant foundation species along much of the wave-exposed rocky coastline of the northeast Atlantic^15–17^, where it forms extensive kelp forest habitat^18,19^, supports high levels of biodiversity^20^ and stores and releases vast amounts of organic matter^21,22^. Despite the critical importance of *L. hyperborea* and the kelp forest habitats it creates in the northeast Atlantic, several important knowledge gaps pertaining to its biology and ecology still persist, including reliable estimates of NPP from multiple populations and across large spatial scales. It is clear, however, that *L. hyperborea* is a ‘season anticipator’ (*sensu*^23^) and exhibits peak growth rates in late winter through spring, when ambient nutrient levels are highest and not limiting^24,25^, and that the species has a cool-temperate distribution, ranging from the Iberian Peninsula polewards to northern Norway and Iceland^16,26^.

**Figure 1:**
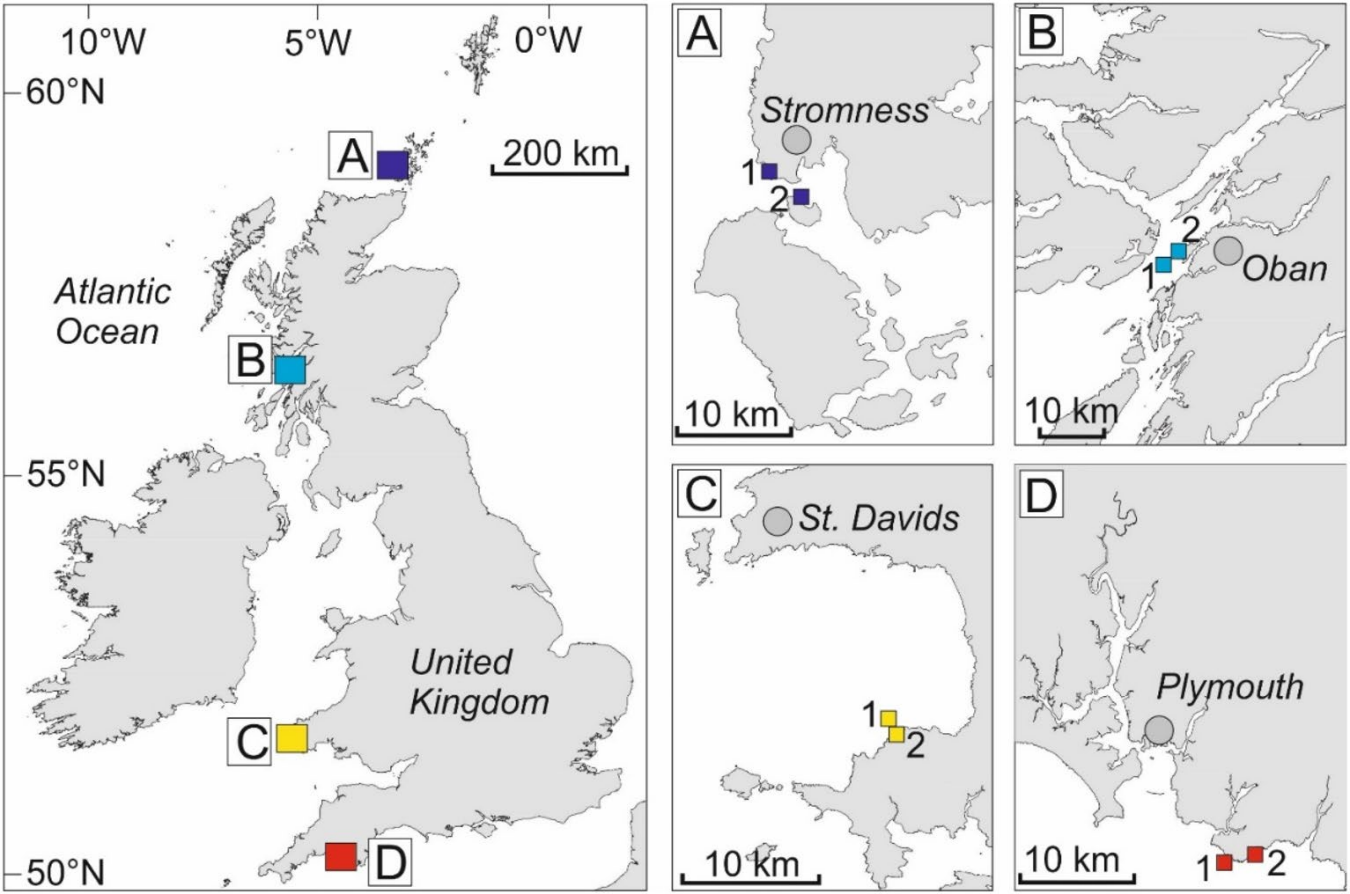
Position of study locations and sites along the UK coastline. Left-hand map shows locations (**A** = north Scotland, **B** = west Scotland, **C** = southwest Wales, **D** = southwest England), right-hand inset maps shows location of each study site within each location. Map produced with ArcGIS 10.3 software (https://www.esri.com/en-us/arcgis/products/index).

We conducted a range of surveys, collections and experiments at eight sites in the UK (Fig. 1), across three years, to examine the role of environmental variables on biomass accumulation and storage within kelp forests. Paired sites, which captured a gradient in wave exposure, were situated within four locations that spanned 9° in latitude (Fig. 1). Crucially, four of the sites (i.e. A1, A2, B1, B2) were located within two ‘cold’ regions (i.e. north and west Scotland), which were on average ~ 2.5 °C cooler than four sites (i.e. C1, C2, D1, D2) located within two ‘warm’ regions (i.e. southwest Wales and southwest England). As such, this large-scale temperature gradient (shown in Table 1 and detailed further in refs^18,21^) allowed us to examine the influence of ocean climate on NPP and standing stock within kelp forests. We also measured a suite of other environmental and ecological variables at each site that may influence rates of NPP (Table 1). Biomass accumulation (as a proxy for NPP) was measured in two different ways. First, we quantified biomass accumulation related to lamina extension, which is the primary component of growth in canopy-forming plants of *L. hyperborea*^27^, which is a long-lived perennial kelp species. Second, we monitored regrowth into cleared areas of forest over three years, to examine maximum potential biomass accumulation during recovery. We also quantified the standing stock of carbon held within kelp populations at each site. Our overall aim was to examine spatial variability in NPP and standing stock, and to link variability patterns with putative environmental drivers, such as ocean temperature, wave exposure, light levels, nutrient availability and sea urchin grazing.

**Table 1:** Summary of environmental and ecological variables measured at each site.

Location	Site	Depth (m BCD)	Spring min T (°C)	Summer max T (°C)	Mean daily light (lumens m^-2^)	Max wave water motion (m s^−1^)	Max tidal water motion (m s^−1^)	Mean NO_3_− + NO_2_− (μM)	Mean PO_4_^3-^ (μM)	Mean density *Echinus* (inds m^−2^)
N Scotland (A)	A1	2	7.06	13.78	3,290	1.32	0.20	2.05	0.22	0.0
N Scotland (A)	A2	4	7.06	13.89	2,592	0.30	0.23	1.92	0.23	0.5
W Scotland (B)	B1	5	7.29	13.99	2,543	0.45	0.15	2.65	0.27	0.1
W Scotland (B)	B2	4	7.21	13.67	1696	0.13	0.05	2.53	0.26	0.1
SW Wales (C)	C1	4	9.97	17.13	1519	0.73	0.21	3.16	0.21	0.2
SW Wales (C)	C2	3	9.29	17.06	976	0.39	0.10	2.67	0.22	0.3
SW England (D)	D1	3	9.65	17.83	1,500	0.89	0.16	3.41	0.13	0.1
SW England (D)	D2	4	10.02	18.10	1553	0.39	0.12	3.05	0.15	0.0

## Results

**Figure 2:**
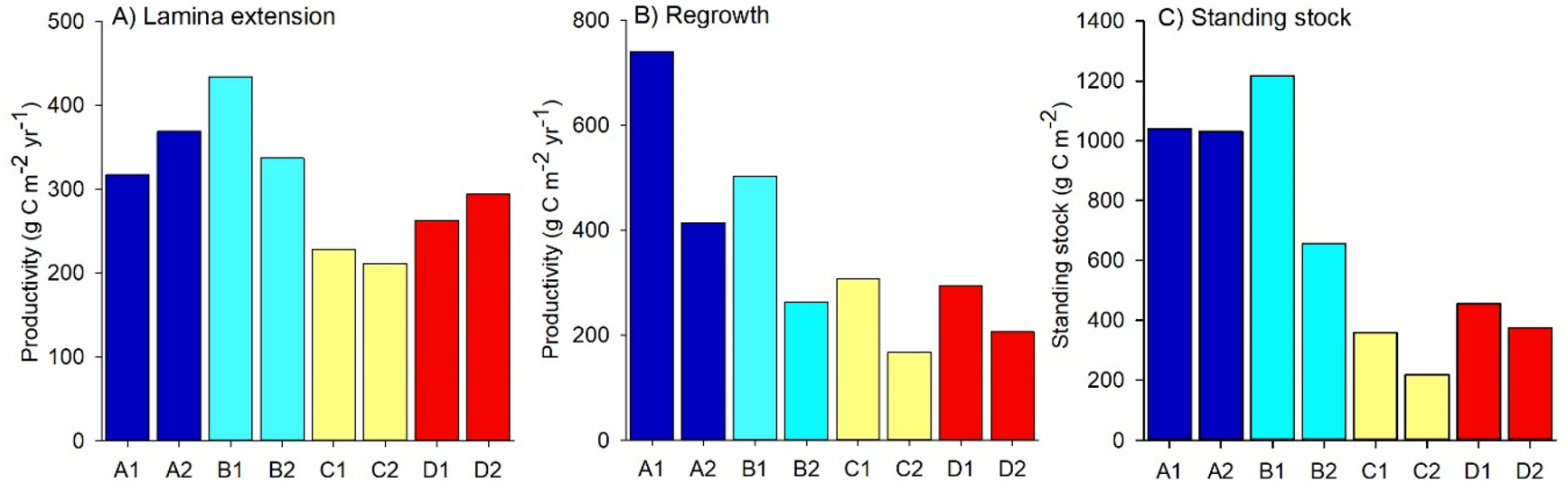
Site-level estimates for NPP relating to (**A**) lamina extension and (**B**) regrowth into cleared areas and for (**C**) carbon standing stock.

Measures of NPP—relating to both lamina extension and regrowth into cleared areas—and standing stock of carbon varied considerably between sites (Fig. 2). For NPP related to lamina extension, the greatest variability was observed between sites in different locations (Fig. 2). Specifically, lamina extension at site B1 was more than twice that at site C2 (434 compared to 211 g C m^-2^ yr^-1^). Variation in NPP related to lamina extension was largely driven by differences in actual biomass accumulation rather than differences in elongation rate or plant density (Fig. S1). For biomass accumulation related to regrowth into cleared areas, much greater variability was observed between sites within the same locations, with rates at the more wave-exposed site consistently higher than the more sheltered site (Fig. 2). Across all eight sites, we recorded a ~ sixfold difference in NPP from regrowth, with the lowest value being 166 g C m^-2^ yr^-1^ at C2 and the highest value being 738 g C m^-2^ yr^-1^ at A1. Variability in NPP from regrowth was largely driven by differences in individual plant biomass, rather than plant density in plots (Fig. S2). Standing stock of carbon was also highly variable (Fig. 2), and ranged from 217 g C m^-2^ at C2 to 1,217 g C m^-2^ at B1. Variation in carbon standing stock was largely driven by differences in plant biomass, rather than density, across sites (Fig. S1).

**Figure 3:**
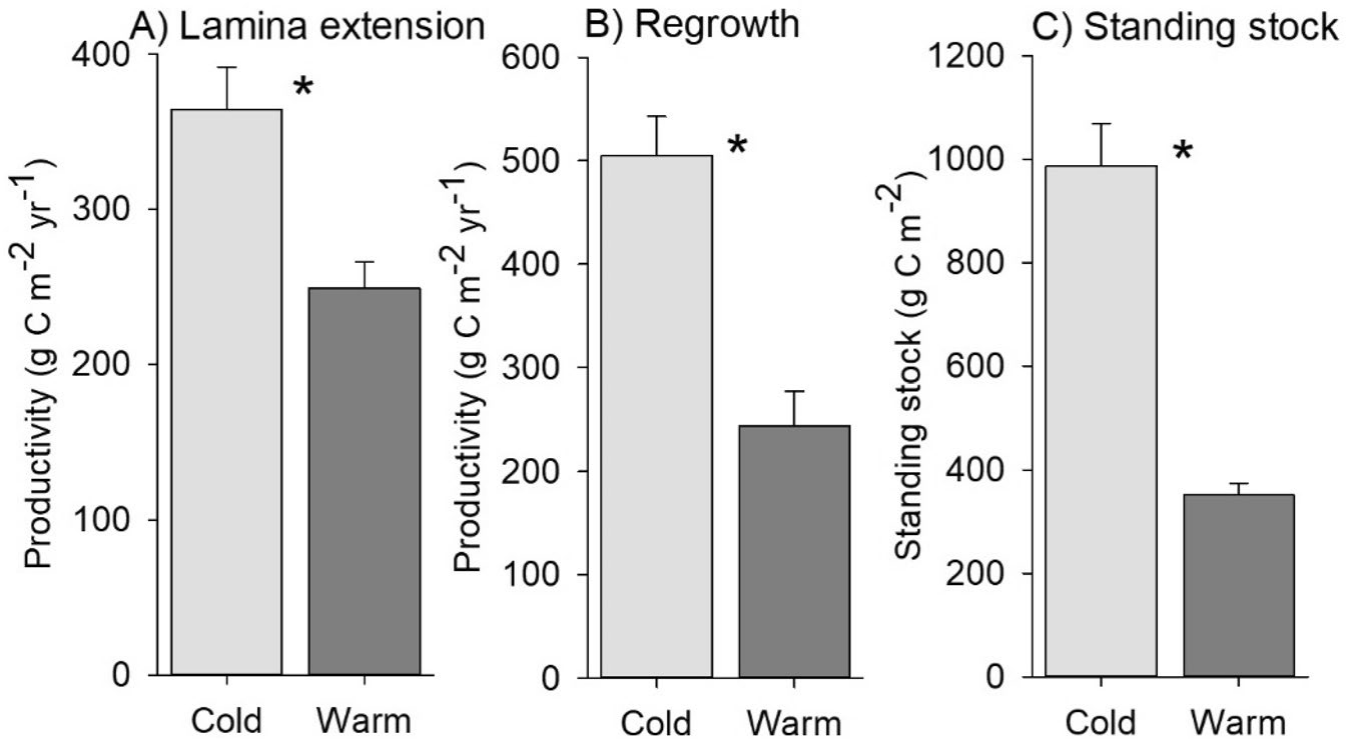
Mean (± SE) values for NPP relating to (**A**) lamina extension and (**B**) regrowth into cleared areas and for (**C**) carbon standing stock for ‘cold’ sites (i.e. A1, A2, B1, B2) versus ‘warm’ sites (i.e. C1, C2, D1, D2). An asterisk indicates significant differences between the cold and warm sites.

Linear models indicated a clear effect of ocean climate, in that all three carbon responses were higher in cold compared to warm waters (Table 2), which was clearly reflected in mean values for northern and southern locations (Fig. 3). Specifically, mean values for colder locations were ~ 1.5, 2, and 3 times greater than the warmer locations for lamina extension, regrowth into cleared areas and standing stock, respectively.

**Table 2:** Contrasts in productivity responses between warm and cold regions and between moderately exposed and exposed sites within each location shown by linear model parameter values.

Responses	Intercept		Climate (warm-cold)			Wave exposure (Moderately exposed – exposed)			Fit	
	a	SE	b1	SE	P	b2	SE	P	Adj R^2^	P
Lamina extension	**368.2**	29.8	**− 115.2**	34.4	0.020	**− **7.9	34.4	0.828	0.57	0.052
Regrowth	**578.7**	63.8	**− 236.3**	73.7	0.024	**− 197.9**	73.7	0.044	0.69	0.023
Standing stock	**1,085.5**	93.8	**− 635.1**	108.4	0.002	**− **197.9	108.4	0.127	0.84	0.005

**Figure 4:**
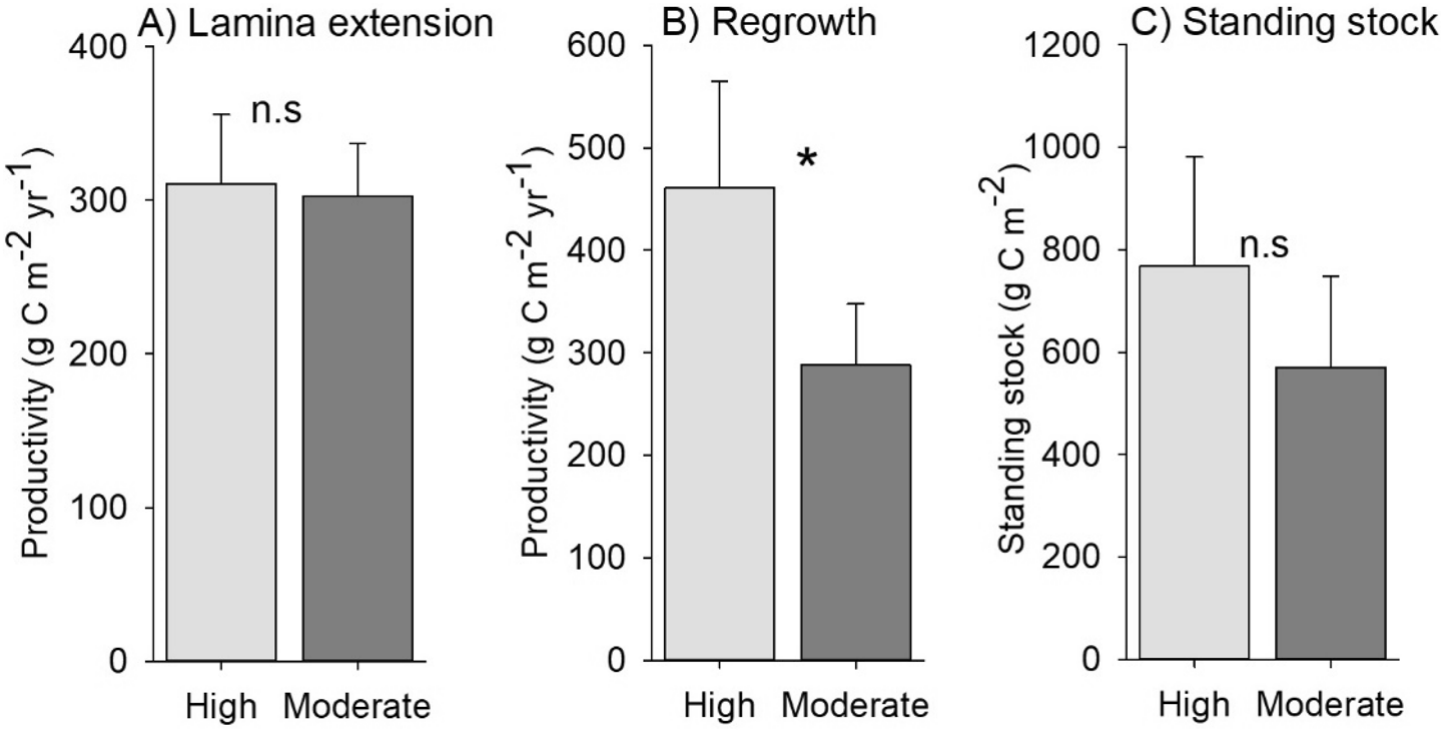
Mean (± SE) values for NPP relating to (**A**) lamina extension and (**B**) regrowth into cleared areas and for (**C**) carbon standing stock for high wave exposure sites versus moderate wave exposure sites (‘n.s.’ indicates non-significant differences).

Regrowth into cleared plots was greater at highly exposed compared to moderately exposed sites, whereas no effect on lamina extension or standing stock was observed (Table 2). Mean values for NPP related to lamina extension were very similar between levels of wave exposure (Fig. 4). Values for NPP related to regrowth and standing stock of carbon differed somewhat between levels of wave exposure with values being 1.6 and 1.3 times greater at high exposure sites, respectively (Fig. 4).

**Figure 5:**
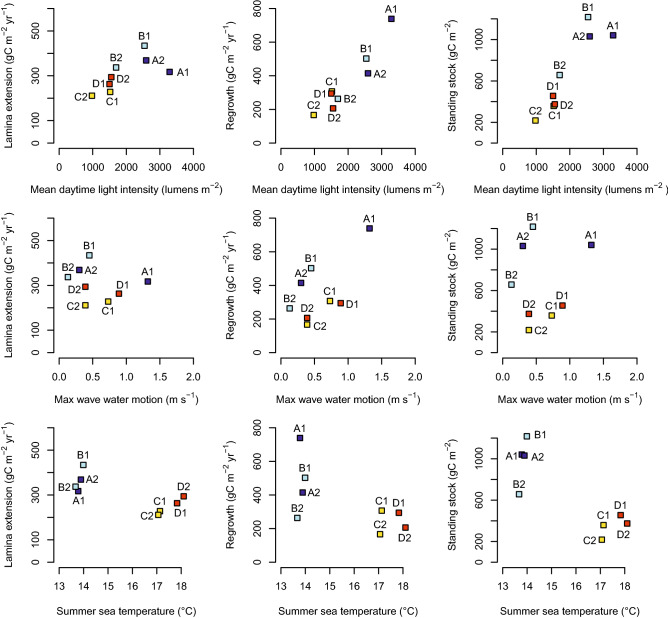
Scatterplots showing relationships between carbon-related variables and key environmental variables (see Table S1). Symbols are colour-coded by location as shown in Fig. 1.

Finally, we examined correlative relationships between the environmental parameters and carbon-related variables at each site (Table S1). All three carbon responses were negatively correlated with summer maximum temperature and positive correlated with light availability (Table S1, Fig. 5; *P* < 0.05). Regrowth into cleared areas was positively but weakly correlated with wave-induced water motion (Table S1, Fig. 5; *P* < 0.10).

## Discussion

Patterns of NPP and standing stock of carbon exhibited high spatial variability, suggesting that the productivity and general functioning of *L. hyperborea* forests is influenced by environmental or ecological factors that vary across similar scales. Overall, our estimates of NPP were comparable to other values reported for *L. hyperborea* across its distribution in the northeast Atlantic (Table 3). Across our sites, the average rate of NPP derived from lamina extension measurements was 306 ± 26 (SE) g C m^-2^ yr^-1^ and from regrowth into cleared plots was 374 ± 64 (SE) g C m^-2^ yr^-1^. The maximum rates we observed were 434 g C m^-2^ yr^-1^ for lamina extension (at site B1) and 738 g C m^-2^ yr^-1^ for regrowth (at site A1). These values are comparable to those recorded for *Macrocystis pyrifera* (‘giant kelp’) forests in California^28^ and globally-distributed estimates of NPP for seagrass meadows^4^. Our maximum values are towards the upper limit for global estimates of maximal NPP derived from macroalgal habitats^9^, indicating the high productivity of *Laminaria hyperborea* stands, particularly in our northernmost locations.

**Table 3:** Rates of primary productivity reported for *Laminaria hyperborea*.

Region	Location	Lat/long	No. of populations	Depth range (m BCD)	Productivity (g C m^-2^ yr^-1^)	Reference
West Scotland	Arisaig	56.9501, −5.8666	2	3–9	360	^24^
West Scotland	Muldoanich	56.9167, −7.4333	5	4–14	279	^27^
West Scotland	Oban	56.4558, −5.4458	3	4–9	197	^27^
West Scotland	Luing	56.2667, −5.6333	2	6–7	561	^27^
Northern Irish Sea	Isle of Mann	54.0858, −4.7700	2	1–2	603	^27^
Southwest England	Plymouth	50.3333, −4.1333	2	0–1	226	^26^
Germany	Heligoland	54.1891, 7.8785	1	0–4	434	^73^
Norway	Hordaland	60.1666, 5.0000	3	3–9	628	^74^
Norway	Vega	65.6766, 11.6410	3	3–9	366	^74^
Norway	Finmark	70.26–224.1852	3	3–9	260	^74^
Iceland	Oddbjarnarsker	65.3443, −23.1723	2	1–19	911	^75^
Brittany	Sark Island	49.4363, −2.3762	1	2	589	^25^
Northern Scotland	Orkney	58.9388, −3.2927	2	2–4	459*	This study
West Scotland	Oban	56.3661, −5.6208	2	4–5	409*	This study
Southwest Wales	Broadhaven	51.7738, −5.1752	2	3–4	228*	This study
Southwest England	Plymouth	50.2911, −4.0472	2	3–4	264*	This study

*Values are averages of the two sites and two methods (lamina extension and regrowth) used within each location.

Light intensity was identified as a putative driver of biomass accumulation and storage, being strongly positively correlated with both NPP (as measured by regrowth into cleared areas) and standing stock. A strong influence of light intensity—which can vary with depth, season, sediment loading and water quality—on kelp growth rates and productivity has been documented in other regions^29–31^ and is explained by the requirement for photosynthetically active radiation (PAR, which is generally correlated with total light intensity^32^) for photosynthesis and growth. Here, NPP and carbon standing stock were notably low at our most turbid site (C2) and very high at our most illuminated site (A1), suggesting an important role of light availability. In UK nearshore waters water clarity (and thus light availability) is highly variable^33^, being influenced by coastal run-off, resuspension of sediments during storms, and pelagic primary productivity^34,35^. There are very few long-term time series on turbidity in UK waters, and those that have been analysed have documented high interannual variability but no clear trends^34,36^. There is, however, a strong link between storm activity and water clarity, with high wind speeds and intense precipitation events leading to reduced light penetration in coastal waters^36^. As such, any future reductions in water clarity driven by increased storm activity may reduce light availability in shallow benthic habitats and affect kelp primary productivity.

By measuring rates of NPP and standing stock across a latitudinal temperature gradient with a consistent methodology, we were able to elucidate the influence of ocean temperature on kelp forest productivity. Our two northernmost locations (i.e. north and west Scotland) were, on average, ~ 2.5 °C colder than the two southernmost locations (i.e. southwest Wales and southwest England) and NPP and carbon standing stock were markedly higher in the colder locations, despite differences in wave exposure and other factors between sites within locations. NPP (estimated from lamina extension) and standing stock were also strongly negatively correlated with spring minimum and summer maximum temperatures. Previous work has shown that population structure^22^ (i.e. plant size and biomass) and detritus production^21^ also vary markedly along this gradient of latitude and temperature; the current study adds to this body of evidence by empirically describing patterns of NPP with temperature. *L. hyperborea* is a cool water species with a latitudinal distribution from its equatorward limit on the Iberian Peninsula through to the high Arctic^16^. The upper thermal limit for survival of *L. hyperborea* is ~ 21 °C^37,38^ with optimal performance at 10–15 °C^39^. As such, temperatures experienced at the southernmost locations exceed thermal optima during some of the year, whereas temperatures in the northernmost locations remain optimal^18,21^. This temperature gradient is likely to drive the observed variability in plant size and biomass^22^ and NPP and carbon standing stock across the latitudinal range considered here, with higher biomass and productivity values in colder waters. The strong influence of ocean temperature on carbon dynamics within these kelp forests is important within the context of climate change, given that increases in temperature have been observed and are predicted for the northeast Atlantic. Without local plasticity or adaptation^40^, we can expect populations in colder locations to perform more similarly to those in the warmer locations in the coming decades, with consequent reductions in NPP and carbon standing stock at the habitat-scale.

Our survey design allowed us to broadly examine the role of wave exposure on NPP and carbon standing stock, as within each location our paired sites were either moderately or fully exposed to wave action. We did not observe ecologically meaningful differences between levels of wave exposure, and carbon dynamics were unrelated to water motion. That said, NPP as measured by regrowth into cleared areas and carbon standing stock were notably higher at wave exposed sites, and regrowth was weakly related to water motion (R^2^ = 0.42, *P* = 0.08). As such, wave exposure and water motion driven by waves may be of some importance, but not as influential as either temperature or light availability across the sites examined here. High water motion can increase growth and productivity in *L. hyperborea* by enhancing diffusion across the surface of the lamina^41^ and by reducing realised intraspecific competition for light^42^. Certainly, the density of plants in cleared plots was highest at the most wave exposed sites (authors’ pers obs) and reductions in realised intraspecific competition may be particularly important in the early stages of recovery into disturbed areas^43^. It is likely that wave exposure is an important factor influencing patterns of NPP in *Laminaria* forests, but the gradient examined in this study was perhaps not broad enough to capture this variability.

Similarly, other factors that we measured—particularly nutrient levels and density of sea urchins—are well-known drivers of kelp population structure and productivity in other regions^44–47^. In UK coastal waters however, nutrient availability is relatively high for much of the spring growth period of *L. hyperborea*^48^. Similarly, the dominant sea urchin, *Echinus esculentus*, does not typically overgraze kelp stands to form urchin barrens, as it is omnivorous and feeds heavily on drift algae and kelp-associated invertebrates^49^. Although overgrazing of *L. hyperborea* by urchins is an important process further north in parts of Norway^50,51^, the principal grazer in that system is *Strongylocentrotus droebachiensis*, which is very rare in the UK as waters are generally too warm for reproduction^52^. While the urchin *E. esculentus* may be important as a consumer of drifting kelp fragments at our study locations, which are plentiful^21^, it is unlikely to exert strong top down pressure on standing stock or NPP. Another factor known to influence the growth of kelp plants is inter-specific competition^53^. However, all of our sites were dominated by mono-specific stands of *L. hyperborea*^18^ and, as such, competition with other macroalgae was likely to have been unimportant.

Our study does have several caveats and limitations that require exploration. First, obtaining in situ growth measurements from subtidal kelp populations is logistically challenging and intensive in terms of time and resources. It was only possible to obtain reliable estimates from eight sites around the UK and—while we believe them to representative of the wider region—it is clear that additional sites encompassing a wider range of environmental conditions would be advantageous. Where possible, further work should incorporate sites that span broader gradients of depth and wave exposure to further elucidate the importance of light availability and water motion, respectively. Second, as our two measures of NPP yielded similar but not identical estimates of productivity rates, it is prudent to examine their usefulness further. NPP estimates based on lamina extension alone do not, by definition, capture production allocated to the stipe and holdfast. However, in mature plants (i.e. > 5 years old), the vast majority of growth (i.e. > 90%) is allocated to the lamina^54^ and, given the age structure of populations at these sites^22^, it is likely that most biomass accumulation was captured. Similarly, we only examined biomass accumulation in mature canopy-forming plants and did not include density or growth rates of subcanopy plants in our estimates of NPP. While subcanopy plants are present at all our sites, sometimes in high densities^55^, they exhibit comparatively low standing biomass^33^ and typically have low rates of biomass accumulation due to reduced light availability beneath dense canopies^56^. Although our values could therefore be underestimates, they are likely to be representative of actual NPP within these kelp forests. Our other approach to measuring NPP involved monitoring regrowth into cleared areas. This method does incorporate growth allocated to all parts of plants and also captures the vast majority of individuals, which largely belong to the same cohort following recruitment. Growth rates may, however, be more strongly influenced by early-successional processes such as recruitment dynamics, intense intra-specific competition and high growth rates of younger plants compared with longer-lived mature plants. As such, NPP rates of younger stands may be higher than that of mature stands. Finally, neither method captured the true amount of carbon assimilated by plants, as a fraction of it (up to 26% in *L. hyperborea*^57^), is exuded as dissolved organic carbon (DOC) and is therefore not accounted for in biomass accumulation estimates. Our overall values for NPP are therefore very likely to be underestimates.

In conclusion, our study has documented rates of NPP and carbon standing stock within *L. hyperborea* forests in UK coastal waters and identified key putative drivers of carbon dynamics. It is clear that, in addition to serving as an important habitat-forming foundation species that supports high biodiversity, the high productivity of these kelp populations is important for inshore carbon cycling and coastal food webs. The estimated total areal extent of *L. hyperborea* in the northeast Atlantic^21^ is around 18,000 km^2^; by taking the average NPP value across studies conducted to date (423 g C m^-2^ yr^-1^, Table 3), we estimate that total biomass accumulation is at least ~ 7.61 Tg C each year across its distributional range. As only a small fraction of this organic matter is consumed directly by grazers, the vast majority of this biomass is released as detritus^55,58^, which may be transported very long distances from source and play an important role within natural carbon sequestration^9,59^ or as a trophic subsidy in adjacent or distant habitats^11,60^. Further work is needed to understand carbon dynamics at the habitat or ecosystem scale (i.e. by quantifying production rates for other macroalgal species and consumption and respiration rates of kelp-associated fauna), which is far better understood in some other kelp forest ecosystems^47,61^. Even so, it is clear that the is flux of carbon through *Laminaria* forests is likely to be significant and has been largely overlooked to date.

## Materials and methods

### Study sites

We quantified rates of NPP and standing stock of carbon for populations of *Laminaria hyperborea* at multiple subtidal rocky reef sites situated along a gradient of ~ 9° of latitude in the northeast Atlantic. Study site pairs were between ~ 180 and 500 km apart (Fig. 1). Within each location a set of candidate study sites were selected based on the following criteria: (i) sites should include sufficient areas of subtidal rocky reef at ~ 2–6 m depth (below chart datum); (ii) sites should be representative of the wider region (in terms of coastal geomorphology) and not obviously influenced by localised anthropogenic activities (e.g. sewage outfalls, fish farms); and (iii) sites should be ‘open coast’ and moderately to fully exposed to wave action to ensure a dominance of *L. hyperborea*. Additionally, our aim was to capture a gradient in wave exposure to examine the influence of hydrodynamics on NPP. Within each location, we selected a ‘high’ and ‘moderate’ exposure site, based on the wave fetch model developed by ref.^62^. The two sites selected within each location were situated between ~ 1 and ~ 5 km apart.

### Measures of primary productivity and standing stock

Kelp forests were characterized by dense stands of *L. hyperborea*, which was the dominant macroalgal species and primary habitat former at all sites (see Ref.^18^ for details on kelp forest structure). We quantified productivity with two proxy measures of NPP – growth associated with extension of lamina and regrowth into cleared plots – and also quantified the standing stock of carbon at each site. While our surveys and experiments were conducted over a relatively short timeframe (i.e. 2–3 years, see below) and did not capture multi-annual or decadal variability in NPP and environmental variables, *L. hyperborea* stands are considered temporally stable^23,63^, particularly in areas protected from overgrazing by urchins, direct harvesting and temperature events which exceed thermal thresholds, and we consider the patterns described here to be representative.

### Lamina extension

For productivity associated with extension of the lamina (hereafter ‘lamina extension’) we used a hole-punch method modified after Ref.^64^. The method involves punching holes at set distances from the stipe/lamina transition zone, where the primary meristem occurs, to capture extension of the lamina over time. In early spring 2015, during the period of peak lamina extension rates for *L. hyperborea*^23^, 15 mature kelp plants (stipe length ≥ 40 cm) were randomly selected, tagged and uniquely labelled at each site by SCUBA divers. For each plant, two holes were punched in the central digit; the first was 5 cm from the base of the lamina, the second 10 cm. After 5–6 weeks, tagged kelps were relocated, harvested and returned to the laboratory for analysis. Some individuals were not recovered or were damaged; 12 replicate plants from each site were selected for further processing. The position of the holes, the fresh weight (FW) of the stipe and lamina were recorded for each plant. To convert elongation rates of lamina tissue (cm) to biomass accumulation (g), three 5 cm-wide segments from the basal parts of the lamina of each plant were cut across their width, cleaned of epiphytes, and weighed (FW biomass). The segment of maximum biomass was then used to calculate biomass accumulation (BA) associated with lamina extension during the observational period, as BA = *g*FW/5*t*, where *g* is observed lamina extension (i.e. the sum of the change in positions of the two punched holes, in cm), FW is the fresh weight (g) of the heaviest strip, and *t* is the number of days between punching the holes and collecting the kelp (e.g.^58,65,66^).

Lamina extension experiments were initiated in late March and ran until early May. In order to convert daily BA observed during the 5–6 weeks of measurements obtained during the peak growth season to annual productivity rates, it was necessary to extrapolate from the spring-time experiment to an annual growth cycle. To do this, we monitored lamina extension rates of two independent populations of *Laminaria hyperborea* in southwest England (location D) each month over an annual cycle, using the hole punch technique described above. Patterns of seasonal variability in lamina extension are presented in detail in Pessarrodona et al.^26^; briefly, as the main growth season (92 days, from March–May) accounts for ~ 79% of total BA observed throughout the year, our daily measurements of lamina extension were extrapolated to annual estimates with: yearly BA = daily BA × 92/79) × 100. The strong seasonality of lamina extension in *Laminaria hyperborea* is characteristic of the species and has been observed in many populations^23^. We also conducted the hole-punch method during the low growth period in summer (July–August 2015) at our study populations along the latitudinal gradient and recorded minimal lamina extension, which aligned with our monthly sampling in southwest England, providing further support for consistency across populations. While BA associated with lamina extension alone does not represent total primary production (i.e. some energy is allocated to growth of the stipe/holdfast and production of storage compounds and DOC), in mature canopy-forming *L. hyperborea* plants the vast majority of growth occurs in the lamina^54,58^.

### Regrowth into cleared areas

We also examined patterns of biomass accumulation into recently cleared plots, as a proxy for maximum potential NPP. Monitoring regrowth of vegetation into deforested areas has frequently been conducted in both terrestrial and marine ecosystems (e.g.^67,68–70^), and is a useful approach for quantifying biomass accumulation within a defined area. In summer 2014, we established two large (~ 6 m diameter) replicate clearings within the kelp forest at each site. SCUBA divers removed all kelp plants within each ~ 28 m^2^ circular plot by hand; plots were then revisited ~ 5 weeks later to remove all smaller plants and any that had been missed during the initial clearing (see example plot in Fig S3). Plots were situated ~ 20 m apart from one another and were fixed using subsurface marker buoys and GPS. The plots were intended to remove competition from canopy forming plants and allow for maximum growth rates for recruits, rather than simulate large-scale disturbances generated by overgrazing by urchins or direct harvesting, for example. After 1 year, young *Laminaria hyperborea* plants had recruited into the plots at high densities, although the small size of plants (stipe length ~ 5–20 cm) led to low standing biomass. After this first year of recruitment, plots were monitored for two years of recovery and, by summer 2017, dense mono-specific stands of large *Laminaria hyperborea* plants had recolonised each plot. The density of plants was quantified by haphazardly placing four 1 m^2^ quadrats within the plot (avoiding the outer 0.5 m perimeter to remove any edge effects) and biomass estimates were obtained by randomly selecting 10 plants from within each plot and returning them to the lab for analysis (i.e. FW biomass). Mean density of plants was multiplied by mean FW biomass and divided by the 2-year post-recruitment recovery period to provide an estimate of annual BA for each plot. Finally, plots were averaged to yield a site-level value of NPP relating to regrowth into cleared areas.

### Standing stock

To characterize the carbon held within kelp forests (i.e. their carbon standing stock), SCUBA divers carried out surveys at each site in spring (April/May) 2015 and 2016 and summer (August/September) 2014, 2015 and 2016. During each sampling event, the density of *L. hyperborea* was quantified by haphazardly placing eight replicate 1 m^2^ quadrats on hard bedrock and recording the density of mature canopy-forming plants (plants defined sensu Bolton, 2016). To convert density to standing biomass, during three sampling events (spring 2015, summer 2014 and 2016), 15 mature *L. hyperborea* plants (i.e. typical canopy-formers) were randomly sampled by cutting them beneath the holdfast and returning them to the laboratory to measure fresh weight (FW) of the holdfast, stipe and lamina. By sampling kelp plants in different seasons and years, we captured natural variability in kelp standing stock. Sampled plants were spatially dispersed across the site and collected from within the kelp forest (rather than at the edge). Average density was multiplied by average plant biomass to generate estimates of standing stock. During the surveys, the density of sea urchins (exclusively *Echinus esculentus*) was also recorded in each quadrat and included as a putative driver of variability patterns in the subsequent analysis (see below).

### Conversions from fresh weight biomass to carbon biomass

All FW biomass values derived from the methods described above were converted to dry weight (DW) and subsequently to carbon (C) biomass using an additional conversion factor. To calculate FW:DW ratios, individual sections of stipe (~ 10 cm length) and lamina (5 cm strips of both basal and distal material) were removed from > 12 plants in summer 2014 and again in spring 2015. Sections were weighed (FW), dried at ~ 60 °C for at least 48 h and then reweighed (DW), as per Ref.^22^. In total over 400 samples were processed; the correlation between FW and DW was strong (ρ > 0.85, *P* < 0.001) and the average FW to DW conversion factor was 0.19 and 0.21 for lamina and stipe tissue, respectively.

Dry weights were subsequently converted to C content by using a conversion factor of 0.3125. This factor was a yearly average obtained from routinely sampling the two independent kelp populations in southwest England (Table S2) and is similar to many other studies on kelp species (see ref.^22^ for other examples). Sampling was conducted approximately every two months to account for seasonal variability in C content. During each sampling event, three individual mature *L. hyperborea* plants were harvested from each population; tissue was then obtained by sectioning a strip of each kelp lamina along its length (~ 4 cm width). The samples were freeze-dried and ground to a fine powder, before quantifying carbon content with a standard elemental analyser (CHN Analyser, EA1110, CE Instruments Ltd, Wigan).

### Environmental variables

At each study site, an array of environmental sensors was deployed to capture temperature, light and relative water motion data at fine temporal resolutions. All arrays were deployed within a 4-week period in July–August 2014 and retrieved ~ 6 weeks later (hereafter ‘summer’), and deployed again in March/April 2015 and retrieved ~ 5 weeks later (hereafter ‘spring’). To quantify water motion induced by waves or tidal flow, an accelerometer (‘HOBO’ Pendant G Logger) was attached to a small buoy and suspended in the water column near the seafloor to allow free movement in response to water motion. The subsurface buoy was tethered to the seabed by a 0.65 m length of rope attached to a clump weight and the accelerometer recorded its position in three axes every 5 min (see^71^ for similar approach and method validation). A temperature and light level sensor (‘HOBO’ Temperature/Light weatherproof Pendant Data Logger 16 k) was also attached to the buoy and captured data every 15 min. The sensor array was deployed for > 35 days at each site and all kelp plants within a ~ 2 m radius of the array were removed to negate their influence on light and water movement measurements. On retrieval, accelerometer data were converted to relative water motion by extracting movement data in the planes of the x and y axes, and first subtracting the modal average of the whole dataset from each value (to account for any static ‘acceleration’ caused by imprecise attachment of the sensor to the buoy and/or the buoy to the tether, which resulted in the accelerometer not sitting exactly perpendicular to the seabed). Accelerometer data were converted to water motion, following^71^. The water motion data were then used to generate two separate metrics, one for movement induced by tidal flow and another for wave action. For tidal flow, extreme values that were most likely related to wave-driven turbulent water movement were first removed (all values above the 90^th^ percentile). Then the range of water motion values recorded within each 12-h period, which encapsulated ~ 1 complete cycle of ebbing and flowing tide, was calculated and averaged over the deployment. For wave-induced water movement, the average of the three highest-magnitude values recorded (following subtraction of average water motion induced by tides) was calculated for each site. Further details of these measurements are provided in Smale et al.^22^.

Temperature data were extracted and converted to daily average, maximum and minimum temperatures. Only periods where all sensor array deployments overlapped were used to generate temperature metrics that could be reliably compared across sites. While in situ temperature data were collected for short periods in both spring and summer, longer term temperature data (obtained over the 3-year study period) were also collected with a Hobo iButton logger, which was deployed at one site within each location on a heavy weight and maintained throughout. Longer term data indicated that the temperature gradient between locations was well defined and consistent, and that data extracted from the shorter deployments were representative (Fig S4). For light intensity, data for the first 14 days of deployment (before fouling by biofilms and epiphytes had the potential to affect light measurements) were used to generate average summer daytime light levels (between 0800 and 2000 h) for each site. Although mounting a light sensor on a non-stationary platform is not ideal because of variation in orientation to sunlight, data from the accelerometers indicated that light sensors at each site were stationary and horizontally-orientated for most of the logged events. As such, in situ light data were deemed robust for making relative comparisons between study sites. At each site, two independent seawater samples were collected from immediately above the kelp canopy with duplicate 50 ml syringes in both spring and summer. Samples were passed through a 0.2 µm syringe filter and kept on ice without light, before being frozen and analysed (within 2 months) for nutrients using standard analytical techniques (see^48^ and references therein). As two samples represented only a ‘snapshot’ of nutrient conditions at each site, we examined additional data sources to ensure that our values were representative and also to confirm that nutrient availability did not covary with temperature. First, we compared our values from southwest England with nearby year-round sampling conducted as part of the Western Channel Observatory (presented in^48^) and found that annual mean nitrate + nitrite and phosphate levels were very similar. Second, we extracted all nutrient data available on the ICES oceanographic database (https://ocean.ices.dk) for the years 1981–2019, within a 50 km radius of the study regions. Data for both nitrate and phosphate were analysed for winter (Nov-Apr) and summer (June-Sept) separately. Nutrient levels did not covary with temperature/latitude in either season and, instead, most variability was observed within rather than between study regions (Fig S5), which corresponds with previous studies on nutrient concentrations in UK coastal waters^72^.

### Statistical analysis

All environmental and biological measurements were conducted at the site level (i.e. eight independent sites). Variability between locations and sites was assessed visually and examined descriptively. Differences in the carbon response variables (lamina extension, plot regrowth and standing stock) between locations were formally examined with two-way analysis of variance using site-level means in linear models in R. As we held the a priori expectation that kelp populations within cold locations (i.e. north and west Scotland, A and B) would perform differently to those within warm locations (i.e. southwest Wales and southwest England, C and D), and because temperature was a key variable of interest, we included the planned contrast of A&B versus C&D within the model. Models were first fitted including the main effects of climate (A&B vs. C&D) and wave action (1 vs. 2 at each location) and the climate × wave action interaction term. The interaction terms were non-significant for all three carbon responses and all environmental variables (at *P* > 0.30) and, given the otherwise very small number of error degrees of freedom in the full model, were therefore dropped from the final models. Relationships between the environmental variables and the carbon response variables were assessed with simple correlation (Pearson’s ρ; as summer maximum and spring minimum temperatures were strongly co-linear only the former was examined). Environmental variables that were correlated with carbon response variables at *P* < 0.10 were considered of interest and examined further with scatterplots.

## Supplementary information

Supplementary file1.
