# Supplementary material for: Environmental factors influencing primary productivity of the forest-forming kelp Laminaria hyperborea in the northeast Atlantic
Source: Sci Rep. 2020 Jul 22;10:12161. doi: 10.1038/s41598-020-69238-x (PMC7376248; doi:10.1038/s41598-020-69238-x)

**Supplementary online material**

Environmental factors influencing primary productivity of the forest-forming kelp *Laminaria hyperborea* in the northeast Atlantic

Dan A. Smale^1^, Albert Pessarrodona^2^, Nathan King^3^, Michael T. Burrows^4^, Anna Yunnie^5^, Thomas Vance^5^, Pippa Moore^6,7^

^1^Marine Biological Association of the United Kingdom, The Laboratory, Citadel Hill, Plymouth PL1 2PB, UK

^2^UWA Oceans Institute and School of Biological Sciences, University of Western Australia, Crawley, 6009 WA, Australia

^3^School of Ocean Sciences, Bangor University, Menai Bridge, Anglesey, LL59 5AB, UK

^4^Scottish Association for Marine Science, Dunbeg, Oban, Argyll, PA37 1QA, UK

*^5^*PML Applications Ltd, Prospect Place, Plymouth, PL1 3DH, UK

^6^Institute of Biological, Environmental and Rural Sciences, Aberystwyth University, Aberystwyth, SY23 3DA, UK.

^7^Centre for Marine Ecosystems Research, School of Natural Sciences, Edith Cowan University, Joondalup 6027, Western Australia, Australia

*Correspondence: Email: [dansma@mba.ac.uk](mailto:dansma@mba.ac.uk) Phone: +44(0)1752 426274

**Table S1.** Correlative relationships between environmental variables and the carbon response variables measured at each site. Pearson’s correlations (ρ) and associated *P* values (in parentheses) are shown for each combination; correlations with *P* < 0.10 were considered of interest and examined further (shown in bold).

| Response variable | Depth (m BCD) | Summer  max T  (°C) | Mean daily light  (lumens m^-2^) | Max wave water motion (m s-1) | Max tidal water motion (m s-1) | Mean NO_3_- +NO_2_- (μM) | Mean PO_4_^3-^  (μM) | Mean density Echinus (inds m^-2^) |
| --- | --- | --- | --- | --- | --- | --- | --- | --- |
| Lamina extension | 0.52 (0.20) | **-0.75 (0.03)** | **0.69 (0.07)** | -0.22 (0.59) | 0.07 (0.85) | -0.37 (0.36) | 0.54 (0.16) | -0.02 (0.95) |
| Regrowth | -0.30 (0.48) | **-0.66 (0.07)** | **0.95 (<0.01)** | **0.64 (0.08)** | 0.54 (0.16) | -0.58 (0.13) | 0.33 (0.42) | -0.21 (0.65) |
| Standing stock | 0.20 (0.65) | **-0.85 (<0.01)** | **0.89 (<0.01)** | 0.13 (0.75) | 0.36 (0.37) | -0.57 (0.17) | 0.56 (0.14) | 0.03 (0.95) |

**Table S2** Monthly and yearly carbon content (g C · g DW; Mean ± SD) from two independent, long-term *L. hyperborea* populations located within region D. Three plants from each site were sampled on every event. The yearly total average (i.e. 0.3125, in bold) was used to convert dry biomass to carbon biomass throughout the study.

| **Month** | **Site 1** | **Site 2** | **Total average** |
| --- | --- | --- | --- |
| April | 0.3000 ± 0.0071 | 0.2769 ± 0.0085 | 0.2885 ± 0.0143 |
| June | 0.2837 ± 0.0057 | 0.2762 ± 0.0035 | 0.2799 ± 0.0059 |
| July | 0.3140 ± 0.0106 | 0.3087 ± 0.0143 | 0.3113 ± 0.0123 |
| September | 0.3336 ± 0.0086 | 0.3312 ± 0.0074 | 0.3324 ± 0.0077 |
| November | 0.3538 ± 0.0091 | 0.3386 ± 0.0189 | 0.3462 ± 0.0162 |
| January | 0.2910 ± 0.0245 | 0.3046 ± 0.0223 | 0.2978 ± 0.0234 |
| **Year** | 0.3156 ± 0.0278 | 0.3097 ± 0.0270 | **0.3125 ± 0.0273** |

**Fig S1.** Mean (± SE) values for kelp biomass dynamics for each site. (A) Lamina elongation rate recorded during peak growth season (n = 12 plants), (B) biomass accumulation associated with lamina extension (n = 12 plants), (C) density of canopy-forming plants (n = 40 quadrats spread over 5 survey events), and (D) canopy-forming plant biomass (n = 45 plants collected over 3 surveys events).


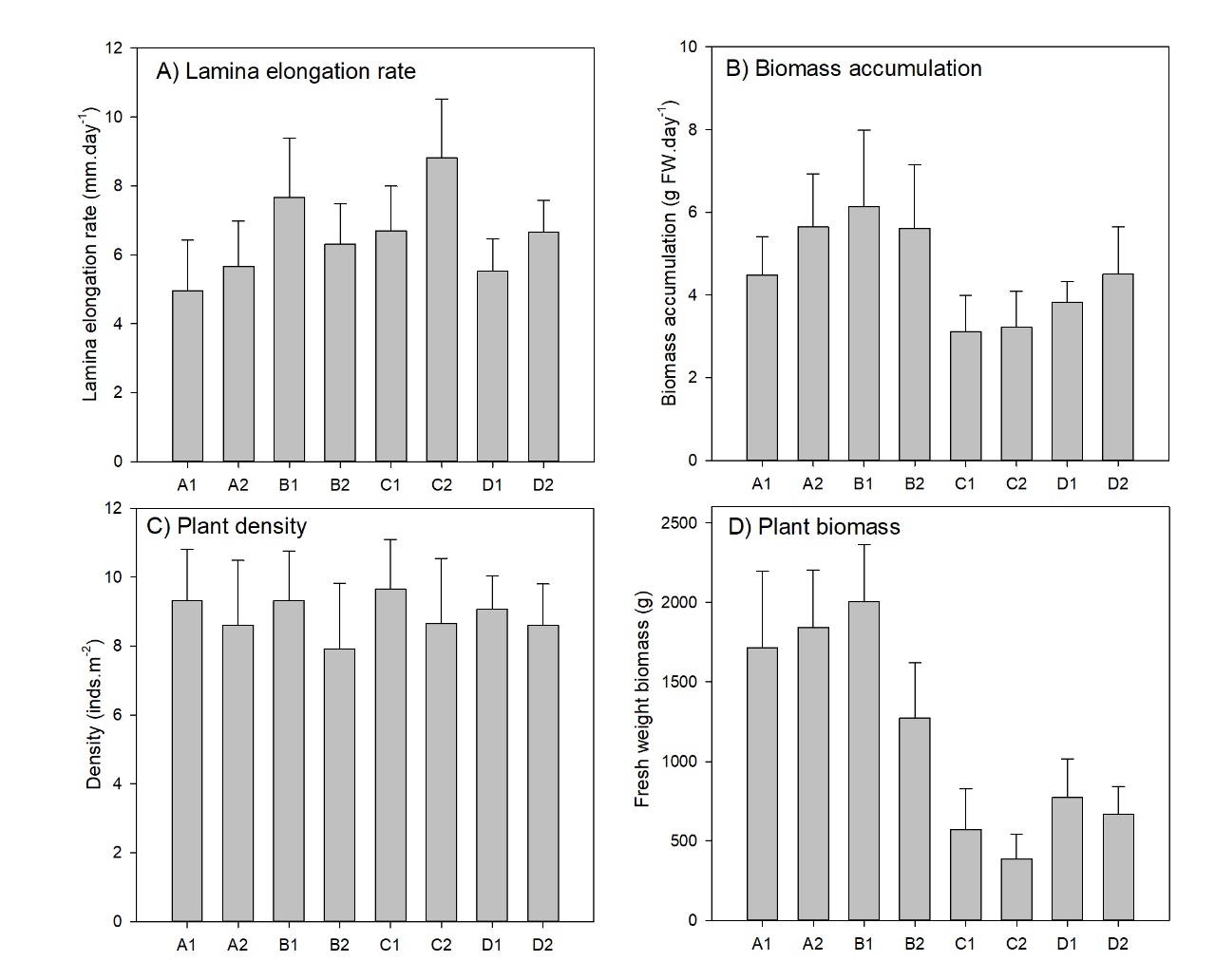


**Fig S2.** Mean (± SE) values for kelp density and plant biomass in clearance plots (3 years post recovery) for each site. (A) Plant density (n = 4 quadrats within each of 2 disturbance plots per site) and (B) plant biomass (n = 5 plants from each of 2 disturbance plots per site).


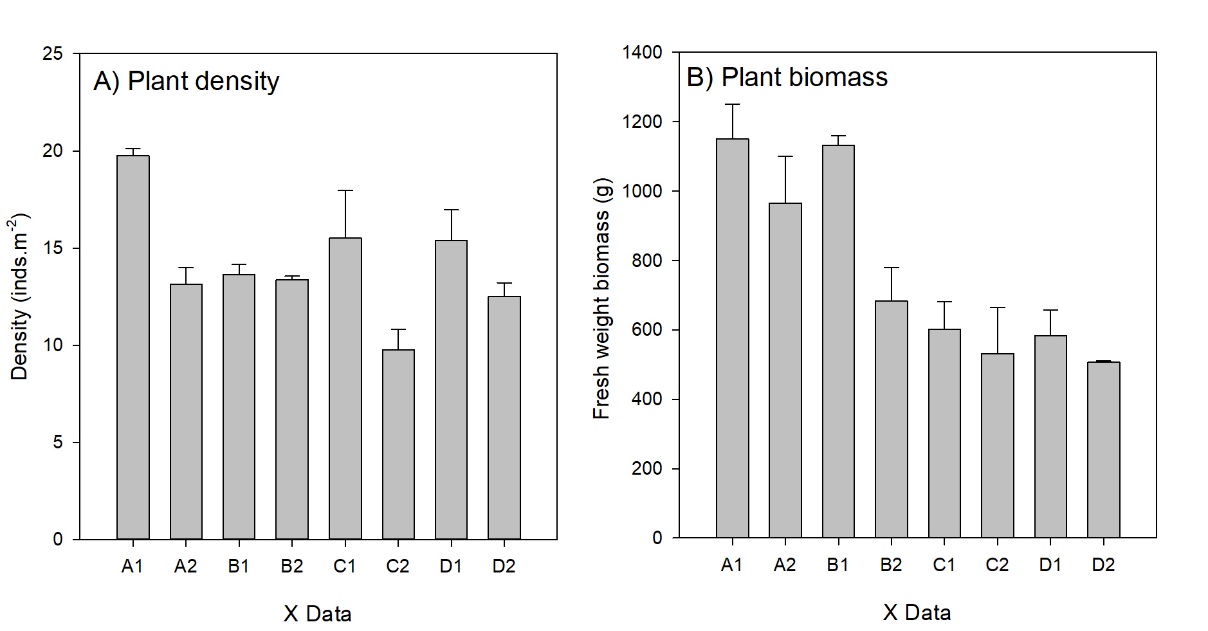

Supplement: Supplementary file 1 — Supplementary file1. [file 41598_2020_69238_MOESM1_ESM.docx]
